# Analysis of Altmetrics in Social Recognition of Neurology and Neurological Disorders

**DOI:** 10.3390/healthcare8040367

**Published:** 2020-09-25

**Authors:** Yerim Kim, Jee-Eun Kim, Sang-Hwa Lee, Dae Young Yoon, Jong Seok Bae

**Affiliations:** 1 Department of Neurology, Kangdong Sacred Heart Hospital, Hallym University College of Medicine, Seoul 05355, Korea; brainyrk@hallym.ac.kr; 2 Department of Neurology, Ewha Womans University Seoul Hospital, Seoul 07804, Korea; junenr@gmail.com; 3 Department of Neurology, Chuncheon Sacred Heart Hospital, Hallym University College of Medicine, Chuncheon 24253, Korea; neurolsh@hallym.or.kr; 4 Department of Radiology, Kangdong Sacred Heart Hospital, Hallym University College of Medicine, Seoul 05355, Korea; daeyoung@kdh.or.kr

**Keywords:** neurology, neurodegenerative diseases, public health informatics, attention, social networking

## Abstract

This study used Altmetric analysis to rank neurological articles and assessed the implications in relation to the social recognition of neurology and neurological disorders. An Altmetric Explorer search was conducted on 25 May 2018 for articles published in the 91 journals included in the 2015 InCites™ Journal Citation Report^®^. We identified and analyzed the 100 articles with the highest Altmetric Attention Scores (AASs). A major proportion of the social impact (high AASs) was focused on neurodegenerative disorders such as dementia and neurodegenerative disorders. About half of the high-ranking articles provided academic information such as disease information (29 articles, 29%), new or advanced treatments (17%), and side effects of treatment (8%). The journal with largest number of top 100 articles was the New England Journal of Medicine (29 articles). Some of the data gathered via altmetrics can change a field of study, the public’s health, or a larger society. This is the first report on the impact of academic articles in neurological disorder on the general public living in our altered information society.

## 1. Introduction

The impact of research articles has traditionally been measured and graded mainly based on the frequency and the numbers of citations during a certain period. Bibliometric analysis utilizing this approach can quantitatively measure the academic influence and the impact of researchers. However, this technique involves a considerable amount of time to have lapsed after articles are published [1]. In addition, certain types of bibliometric analysis have disclosed that specific academic trends or popular issues may contribute to articles being ranked highly [2]. Another crucial issue to consider is that bibliometrics does not reflect the impact of articles on the general public.

The acquisition of medical information is changing markedly with the increasing popularity of the internet. This means that many people are nowadays exposed to medical news from mass communication and social network systems as well as from academic articles written by specialists [3,4]. For example, online platforms such as Twitter, blogs, and online sites of community addressing medical information can rapidly disseminate new information and academic outcomes related to medical science. It is therefore reasonable to assume that this changing environment will alter attitudes toward learning about new medical information.

Several tools have been developed to address the impact of this new communication environment [5,6,7]. Altmetric analysis is one such commonly available tool and is now being used by some influential medical journals for measuring the social impact of their publications. Altmetrics is a term that has been used in the literature to refer to both article-level and alternative metrics. It can measure and visualize the impact of research articles on various new online platforms [8]. Therefore, Altmetric analysis can be used to estimate the dissemination of medical information related to certain research articles.

Neurology is a specialty of clinical medicine that covers numerous subspecialties such as dementia, stroke, epilepsy, demyelinating disorders, parkinsonism, and neuromuscular disorders. These disorders have been among the most popular subjects of investigation in neuroscience research, and many basic research studies related to these diseases are ongoing, which is rapidly improving the understanding of the basic mechanisms underlying the diseases. Neurologists across all subspecialties also perform clinical research based on advances in basic research, and some of their outcomes have been drawn to the attention of the general public thanks to them being highlighted in the mass media. The aim of this study was to determine which recent papers in the field of neurology have received the highest Altmetric Attention Scores (AASs). This study analyzed the highly ranked articles in the fields of neurology and neurological disorders as identified using Altmetric analysis and evaluated the implications of this technique in terms of the recognition of neurology and neurological disorders among the general public.

## 2. Materials and Methods

### 2.1. Study Design and Setting

The web-based application Altmetric Explorer (Altmetric, London, UK) was utilized as a search engine in this study. The results were visualized using the Altmetric donut, which is a multicolored circle that displays the relative contributions of all sources. This donut symbolizes the dissemination of research output via different sources (Facebook, Twitter, Google+, Linked In, Sina Weibo, and Pinterest), and the display format demonstrates a weighted score. The Altmetric Attention Score (AAS) is presented in the center of the graphic. Detailed information about the algorithm and examples of its application are available elsewhere [6,9].

An Altmetric Explorer search was conducted on 25 May 2018 for articles published in the 91 journals included in the 2015 InCites™ Journal Citation Report^®^ grouped under the subject categories of clinical neurology, neuroscience, and general and internal medicine. The site was accessed on only a single specific day (25 May 2018) to avoid changes in the online presentation of articles interfering with the analyses. No restrictions were applied regarding language, date of publication, AAS, keywords, title of output, type of output, or scholarly identifiers.

Data cannot be shared because there are embargoes on datasets. Anonymized data will be shared by request from any qualified investigator.

### 2.2. Data Sources

We analyzed the full text of the 100 articles with the top AASs and extracted detailed information about title, year of publication, AAS, corresponding author, journal name, and journal impact factor (based on the 2017 science edition). Because of considerable subject overlap between articles in the clinical neurology and the neuroscience fields, the authors first analyzed clinical neurology journals and thereafter included additional articles in neuroscience journals that provided clear information regarding neurological disorders. We also analyzed the articles in general and internal medicine journals that focused on neurological disorders as the main subject. After selecting 100 articles, we categorized them into 15 subspecialties of disease groups and 5 categories of main subjects.

Two neurologists (Yerim Kim. and Jong Seok Bae) independently identified and analyzed the articles. In cases of disagreement between these two reviewers, consensus was achieved through open discussions. The present study adopted a descriptive research approach by means of bibliometric analysis.

### 2.3. Standard Protocol Approvals, Registrations, and Patient Consents

The present study did not involve human subjects and thus did not require approval from an institutional review board. We used STROBE guidelines for a descriptive study [10].

## 3. Results

We finally selected the 100 articles with the highest AASs (range = 868–3873), as listed in Table 1. The journal with largest number of top 100 article was the *New England Journal of Medicine* (29 articles), while the *Neurology* journal had the largest number of articles in the field of clinical neurology (13 articles). We investigated the type of access because they can also affect the accessibility of social media (Table 2). The most common author nationality was American (Table 3). In cases of a co-corresponding author, the nationality of the first author was listed.

Regarding the disease category or subspecialty, social recognition was relatively concentrated on degenerative disorders and cerebrovascular disorders. Research articles in the neuroscience field providing advanced information about disease mechanisms had the second highest ranks (Table 4). A particularly interesting finding was that most of the articles classified in the neuroscience field also contained subjects related to cognitive and behavioral science (e.g., memory, addiction, and intelligence). These findings suggest that dementia and cognitive/neurobehavioral problems receive more attention from the general public.

We arbitrarily categorized the articles in the following five subjects based on interest in health and medical services among the general public: disease information, lifestyle and disease, science information, new or advanced treatments, and side effects of treatment. As indicated in Table 5, the ranking order of subject categories was identical to the order in the above list. About half of the high-rank articles provided medical information such as disease information (29 articles, 29%), new or advanced treatments (17%), and side effects of treatment (8%). However, half of the 100 articles were about less academic subjects, with 27 articles (27%) addressing the relationship between lifestyle and diseases.

## 4. Discussion

This Altmetric analysis revealed the current status of social recognition and public attention regarding neurology and neurological disorders. This is the first trial to evaluate this subject based on the premise that the acquisition of information and learning behaviors will eventually change from before the era of easy internet accessibility. Of the various subspecialties in neurology, research into degenerative and cerebrovascular disorders receives the strongest interest from the general public. People are frequently exposed to journals in general and internal medicine and clinical neurology with high impact factors via the present environment of information flow. In addition to the topic of disease information, members of the general public pay considerable attention to the relationship between lifestyle and disease. The present results may be utilized when establishing policies or planning promotions for public health in neurology and neurological disorders.

With the global accessibility and the popularity of the internet and social media, the utility of Altmetric analysis has grown rapidly since the concept was first introduced in 2011 [11]. This trend is expected to continue, because, by 2020, the number of worldwide social media users is expected to reach 2.95 billion, or around one-third of the worldwide population [12]. 

The article with the highest AAS (=3873) was an invited editorial (in the form of a letter) written by the wife of a famous patient [13]. Despite not being a medical specialist, she provided a precise description of the disease course and the treatment of her husband, Robin Williams. According to both his disease course and pathological examination, he tragically ended his life due to severe neuropsychiatric symptoms from diffuse Lewy body disease. The subjects of most of the other top 10 ranked articles were related to environment and lifestyle and diseases, such as chronic trauma, alcohol, living environment, and nutrients [14,15,16,17].

About half of the top 100 ranked articles were related to clinical aspects of medical information, such as disease information (29 articles, 29%), new or advanced treatments (17%), and side effects of treatment (8%). However, the other half of the articles focused on less academic subjects, with 27 articles (27%) addressing the relationship between lifestyle and diseases, as exemplified above. Science information rather than specific disease diagnoses/treatments was the subject in 19 articles (e.g., newly demonstrated mechanisms of memory persistence, the mechanism of dreaming, and the genetics of intelligence) [18,19].

Our list naturally included some diseases that give rise to social problems. One of these is the Zika virus and its neurological complications, such as microcephaly and Guillain-Barré syndrome [20,21]. Another issue is chronic traumatic encephalopathy, which is a major problem among Americans [14]. Many American parents are informed about this issue by studies of cognitive performance and chronic physical head trauma, especially for American football athletes. It can therefore be presumed that the results of Altmetric analysis that are of interest to the general public may change as new social issues develop.

Another distinct characteristic of our analysis is that the articles ranked highly in the Altmetric analysis appeared to use general terminology for disorders in their titles rather than specific disease entities; for example, “Alzheimer’s disease” was less common than “dementia” and “cognitive or intellectual dysfunction/disorders.” This finding implies that common and easy-to-understand terminology might be more acceptable to the general public.

## 5. Conclusions

It is reasonable to use traditional citation-based metrics to measure the impact of a scholarly article in the sense that citations indicate that new evidence has been built upon by the findings of a given paper. Altmetrics, on the other hand, appears to reflect public interest rather than the scientific merits of an article. It should be remembered that there can be considerable discordance between the expectations of academic and medical value among scholars and the attitudes of the general public.

## Figures and Tables

**Table 1:** Top 100 articles with the highest Altmetric Attention Scores (AASs).

Rank	Article Title	AAS	Journal	Corresponding Author	Country
1	The terrorist inside my husband’s brain	3873	Neurology	Susan Schneider Williams	USA
2	Clinicopathological evaluation of chronic traumatic encephalopathy in players of American football	3674	JAMA: Journal of the American Medical Association	Ann C. McKee	USA
3	Dementia prevention, intervention, and care	3294	The Lancet	Gill Livingston	UK
4	Moderate alcohol consumption as risk factor for adverse brain outcomes and cognitive decline: longitudinal cohort study	3222	British Medical Journal	Anya Topiwala	UK
5	Sugar- and artificially sweetened beverages and the risks of incident stroke and dementia	3215	Stroke	Matthew P. Pase	USA
6	Living near major roads and the incidence of dementia, Parkinson’s disease, and multiple sclerosis: a population-based cohort study	2924	The Lancet	Hong Chen	Canada
7	Neurobehavioral effects of developmental toxicity	2870	Lancet Neurology	Philippe Grandjean	USA
8	Nutrients and bioactives in green leafy vegetables and cognitive decline: prospective study	2538	Neurology	Martha Clare Morris	USA
9	Trial of cannabidiol for drug-resistant seizures in the Dravet syndrome	2516	New England Journal of Medicine	Orrin Devinsky	USA
10	Zika virus associated with microcephaly	2400	New England Journal of Medicine	Tatjana Avšič Županc	Slovenia
11	Were James Bond’s drinks shaken because of alcohol induced tremor?	2249	British Medical Journal	Patrick Davies	UK
12	Guillain-Barré Syndrome outbreak associated with Zika virus infection in French Polynesia: a case-control study	2202	The Lancet	Arnaud Fontanet	Francench Polynesia
13	Post-study caffeine administration enhances memory consolidation in humans	2202	Nature Neuroscience	Michael A. Yassa	USA
14	Association between dietary factors and mortality from heart disease, stroke, and type 2 diabetes in the United States	2190	JAMA: Journal of the American Medical Association	Renata Micha	USA
15	Long working hours and risk of coronary heart disease and stroke: a systematic review and meta-analysis of published and unpublished data for 603,838 individuals	2016	The Lancet	Mika Kivimäki	UK
16	Thrombectomy 6 to 24 h after stroke with a mismatch between deficit and infarct	1591	New England Journal of Medicine	Tudor G. Jovin	USA
17	Concussion, microvascular injury, and early tauopathy in young athletes after impact head injury and an impact concussion mouse model	1563	Brain	Lee E. Goldstein	USA
18	Incidence of dementia over three decades in the Framingham Heart Study	1544	New England Journal of Medicine	Sudha Seshadri	USA
19	Zika virus infection with prolonged maternal viremia and fetal brain abnormalities	1537	New England Journal of Medicine	Rita W. Driggers	USA
				Adre du Plessis	Finland
				Olli Vapalahti	Finland
20	Mixed pathologies including chronic traumatic encephalopathy account for dementia in retired association football (soccer) players	1532	Acta Neuropathologica	Janice L. Holton	UK
				Tamas ReveszTamas Revesz	UK
21	Obesity associated with increased brain age from midlife	1491	Neurobiology of Aging	Lisa Ronan	UK
21	Mnemonic training reshapes brain networks to support superior memory	1491	Neuron	Martin Dresler	The Netherlands
23	Mediterranean diet and age-related cognitive decline: a randomized clinical trial	1486	JAMA Internal Medicine	Emilio Ros	Spain
24	Mediterranean-type diet and brain structural change from 73 to 76 years in a Scottish cohort	1471	Neurology	Michelle Luciano	UK
25	Association of proton pump inhibitors with risk of dementia: a pharmacoepidemiological claims data analysis	1458	JAMA Neurology	Britta Haenisch	Germany
26	Thrombectomy for stroke at 6 to 16 h with selection by perfusion imaging	1426	New England Journal of Medicine	Gregory W. Albers	USA
27	Restoration of reaching and grasping movements through brain-controlled muscle stimulation in a person with tetraplegia: a proof-of-concept demonstration	1403	The Lancet	A. Bolu Ajiboye	USA
28	A controlled trial of erenumab for episodic migraine	1378	New England Journal of Medicine	Peter J. Goadsby	UK
29	Dutch courage? Effects of acute alcohol consumption on self-ratings and observer ratings of foreign language skills	1350	Journal of Psychopharmacology	Fritz Renner	The Netherlands
30	Fully implanted brain–computer interface in a locked-in patient with ALS	1333	New England Journal of Medicine	Nick F. Ramsey	The Netherlands
31	Alzheimer’s disease drug-development pipeline: few candidates, frequent failures	1281	Alzheimer’s Research & Therapy	Jeffrey L. Cummings	USA
32	Alcohol acutely enhances decoding of positive emotions and emotional concern for positive stimuli and facilitates the viewing of sexual images	1271	Psychopharmacology	Matthias E. Liechti	Switzerland
33	Regression of glioblastoma after chimeric antigen receptor T-cell therapy	1252	New England Journal of Medicine	Behnam Badie	USA
34	Clinical features and neuroimaging (CT and MRI) findings in presumed Zika virus related congenital infection and microcephaly: retrospective case series study	1249	British Medical Journal	Maria de Fatima Vasco Aragao	Brazil
35	Nuclear receptor NR1H3 in familial multiple sclerosis	1226	Neuron	Weihong Song	Canada
				Carles Vilariño-Güell	Canada
36	Zika and the risk of microcephaly	1225	New England Journal of Medicine	Michael A. Johansson	USA
37	Single-dose gene-replacement therapy for spinal muscular atrophy	1222	New England Journal of Medicine	Jerry R. Mendell	USA
38	Enhancing dentate gyrus function with dietary flavanols improves cognition in older adults	1218	Nature Neuroscience	Scott A. Small	USA
38	Surface-based morphometry reveals the neuroanatomical basis of the five-factor model of personality	1218	Social Cognitive & Affective Neuroscience	Luca Passamonti	UK
40	Bilingualism delays age at onset of dementia, independent of education and immigration status	1209	Neurology	Suvarna Alladi	India
41	Hematopoietic stem-cell gene therapy for cerebral adrenoleukodystrophy	1190	New England Journal of Medicine	David A. Williams	USA
42	Circadian rest-activity pattern changes in aging and preclinical Alzheimer disease	1183	JAMA Neurology	Yo-El S. Ju	USA
43	Cannabinoids for epilepsy—real data, at last	1171	New England Journal of Medicine	Samuel F. Berkovic	Australia
44	Association of playing high school football with cognition and mental health later in life	1153	JAMA Neurology	Dylan S. Small	USA
45	Migraine and vascular disease	1131	British Medical Journal	Rebecca C. Burch	USA
46	Emotional brain states carry over and enhance future memory formation	1123	Nature Neuroscience	Lila Davachi	USA
47	What paint can tell us: a fractal analysis of neurological changes in seven artists	1113	Neuropsychology	Alex Forsythe	UK
48	Evidence of amyloid-β cerebral amyloid angiopathy transmission through neurosurgery	1097	Acta Neuropathologica	Sebastian Brandner	UK
49	Neurobiologic advances from the brain disease model of addiction	1092	New England Journal of Medicine	Nora D. Volkow	USA
50	Zika virus associated with meningoencephalitis	1082	New England Journal of Medicine	Guillaume Carteaux	France
51	Age-specific risks, severity, time course, and outcome of bleeding on long-term antiplatelet treatment after vascular events: a population-based cohort study	1081	The Lancet	Peter M. Rothwell FMedSci	UK
52	Evidence for sugar addiction: behavioral and neurochemical effects of intermittent, excessive sugar intake	1077	Neuroscience & Biobehavioral Reviews	Bartley G. Hoebel	USA
53	MIND diet associated with reduced incidence of Alzheimer’s disease	1074	Alzheimer’s & Dementia	Martha Clare Morris	USA
54	The neural correlates of dreaming	1070	Nature Neuroscience	Giulio Tononi	USA
55	Trial of amitriptyline, topiramate, and placebo for pediatric migraine	1069	New England Journal of Medicine	Scott W. Powers	USA
56	The feasibility of a brain-computer interface functional electrical stimulation system for the restoration of overground walking after paraplegia	1062	Journal of NeuroEngineering and Rehabilitation	An H. Do	USA
				Zoran Nenadic	USA
57	2018 Guidelines for the Early Management of Patients with Acute Ischemic Stroke: a guideline for healthcare professionals from the American Heart Association/American Stroke Association	1061	Stroke	the American Heart Association Stroke Council	USA
58	The persistence and transience of memory	1049	Neuron	Paul W. Frankland	Canada
59	Meditation experience is associated with increased cortical thickness	1042	NeuroReport	Sara W. Lazar	USA
60	Practice guideline update summary: Mild cognitive impairment: Report of the Guideline Development, Dissemination, and Implementation Subcommittee of the American Academy of Neurology	1038	Neurology	American Academy of Neurology	USA
61	Glucose levels and risk of dementia	1020	New England Journal of Medicine	Paul K. Crane	USA
62	Does playing violent video games cause aggression? A longitudinal intervention study	1017	Molecular Psychiatry	Simone Kühn	Germany
63	Changes in sleep duration, quality, and medication use are prospectively associated with health and well-being: analysis of the UK Household Longitudinal Study	1000	Sleep	Nicole K. Y. Tang	UK
64	Vitamin D and the risk of dementia and Alzheimer disease	989	Neurology	David J. Llewellyn	UK
65	Ocrelizumab versus placebo in primary progressive multiple sclerosis	975	New England Journal of Medicine	Xavier Montalban	Spain
66	The busier the better: greater busyness is associated with better cognition	958	Frontiers in Aging Neuroscience	Sara B. Festini	USA
67	Hearing loss and cognitive decline in older adults	956	JAMA Internal Medicine	Frank R. Lin	USA
67	Anxiety cells in a hippocampal-hypothalamic circuit	956	Neuron	Mazen A. Kheirbek	USA
				René Hen	USA
69	Midlife cardiovascular fitness and dementia: a 44-year longitudinal population study in women	954	Neurology	Helena Hörder	Sweden
70	Time to wake up and smell the coffee? Coffee consumption and multiple sclerosis	951	Journal of Neurology, Neurosurgery & Psychiatry	Elaine Kingwell	Canada
71	Alcohol intake and cognitively healthy longevity in community-dwelling adults: the Rancho Bernardo Study	950	Journal of Alzheimer’s Disease	Erin Richard	USA
72	Glioproliferative lesion of the spinal cord as a complication of “stem-cell tourism”	949	New England Journal of Medicine	Aaron L. Berkowitz	USA
				Michael B. Miller	USA
72	Mindfulness meditation and improvement in sleep quality and daytime impairment among older adults with sleep disturbances: a randomized clinical trial	949	JAMA Internal Medicine	David S. Black	USA
74	Preventing intrusive memories after trauma via a brief intervention involving Tetris computer game play in the emergency department: a proof-of-concept randomized controlled trial	944	Molecular Psychiatry	E. A. Holmes	Sweden
75	Cumulative use of strong anticholinergics and incident dementia: a prospective cohort study	942	JAMA Internal Medicine	Shelly L. Gray	USA
76	Sleep and human aging	941	Neuron	Matthew P. Walker	USA
77	A 2 year multidomain intervention of diet, exercise, cognitive training, and vascular risk monitoring versus control to prevent cognitive decline in at-risk elderly people (FINGER): a randomized controlled trial	939	The Lancet	Miia Kivipelto	Sweden
78	Leisure activities and the risk of dementia in the elderly	931	New England Journal of Medicine	Joe Verghese	USA
78	Urinary 8-oxo-7,8-dihydroguanosine as a potential biomarker of aging	931	Frontiers in Aging Neuroscience	Lan-Lan Wang	China
				Jian-Ping Cai	China
80	Poor sleep is associated with CSF biomarkers of amyloid pathology in cognitively normal adults	930	Neurology	Kate E. Sprecher	USA
81	Migraine and risk of cardiovascular disease in women: prospective cohort study	929	British Medical Journal	Tobias Kurth	Germany
82	Video gaming in school children: how much is enough?	918	Annals of Neurology	Jesus Pujol	Spain
83	Incidence of subarachnoid hemorrhage is decreasing together with decreasing smoking rates	913	Neurology	Miikka Korja	Finland
84	Mild TBI and risk of Parkinson disease: A Chronic Effects of Neurotrauma Consortium Study	912	Neurology	Raquel C. Gardner	USA
85	Sauna bathing reduces the risk of stroke in Finnish men and women: a prospective cohort study	911	Neurology	Setor K. Kunutsor	UK
86	Time trends in the incidence of Parkinson disease	910	JAMA Neurology	Walter A. Rocca	USA
86	A randomized controlled trial to test the effect of multispecies probiotics on cognitive reactivity to sad mood	910	Brain, Behavior, & Immunity	Laura Steenbergen	The Netherlands
88	Analgesic effects of alcohol: a systematic review and meta-analysis of controlled experimental studies in healthy participants	906	Journal of Pain	Trevor Thompson	UK
89	Sleep loss promotes astrocytic phagocytosis and microglial activation in mouse cerebral cortex	905	Journal of Neuroscience	Chiara Cirelli	USA
90	A qualitative impairment in face perception in Alzheimer’s disease: evidence from a reduced face inversion effect	903	Journal of Alzheimer’s Disease	Sven Joubert	Canada
91	Acute and chronic effects of cannabinoids on effort-related decision-making and reward learning: an evaluation of the cannabis ‘amotivational’ hypotheses	898	Psychopharmacology	Will Lawn	UK
92	Alcohol consumption and cognitive decline in early old age	895	Neurology	Séverine Sabia	UK
92	Genetics and intelligence differences: five special findings	895	Molecular Psychiatry	R. Plomin	UK
94	Time to treatment with endovascular thrombectomy and outcomes from ischemic stroke: a meta-analysis	888	JAMA: Journal of the American Medical Association	Michael D. Hill	Canada
95	Risk of pneumonia associated with incident benzodiazepine use among community-dwelling adults with Alzheimer disease	884	CMAJ: Canadian Medical Association Journal	Heidi Taipale	Finland
95	Aerobic exercise and vascular cognitive impairment: a randomized controlled trial	884	Neurology	Teresa Liu-Ambrose	Canada
97	Old brains come uncoupled in sleep: slow wave-spindle synchrony, brain atrophy, and forgetting	875	Neuron	Randolph F. Helfrich	USA
98	Neuroscience-inspired artificial intelligence	873	Neuron	Demis Hassabis	UK
98	Seeing Jesus in toast: neural and behavioral correlates of face pareidolia	873	Cortex	Jie Tian	China
				Kang Lee	Canada
100	The cumulative cost of additional wakefulness: dose-response effects on neurobehavioral functions and sleep physiology from chronic sleep restriction and total sleep deprivation	868	Sleep	Hans P.A. Van Dongen	USA

**Table 2:** Journals with top 100 articles, ranked according to the AAS.

Rank	Journal Name	Number of Articles	Type of Access
1	New England Journal of Medicine	20	S
2	Neurology	13	S
3	The Lancet	7	S
3	Neuron	7	S
5	British Medical Journal	5	S
6	Nature Neuroscience	4	S
6	JAMA Neurology	4	S
6	JAMA Internal Medicine	4	S
9	Molecular Psychiatry	3	S
9	JAMA: Journal of the American Medical Association	3	S
11	Stroke	2	S
11	Sleep	2	S
11	Psychopharmacology	2	S
11	Journal of Alzheimer’s Disease	2	S
11	Frontiers in Aging Neuroscience	2	OA
11	Acta Neuropathologica	2	S
18	Neurobiology of Aging	1	S
18	Journal of Psychopharmacology	1	S
18	Alzheimer’s Research & Therapy	1	OA
18	Brain	1	S
18	NeuroReport	1	S
18	Journal of NeuroEngineering and Rehabilitation	1	OA
18	Alzheimer’s & Dementia	1	S
18	Neuroscience & Biobehavioral Reviews	1	S
18	Social Cognitive & Affective Neuroscience	1	OA
18	Neuropsychology	1	S
18	Journal of Neurology Neurosurgery & Psychiatry	1	S
18	Lancet Neurology	1	S
18	Annals of Neurology	1	S
18	Brain Behavior & Immunity	1	S
18	Journal of Pain	1	S
18	Cortex	1	S
18	CMAJ: Canadian Medical Association Journal	1	S
18	Journal of Neuroscience	1	S

Abbreviations: S = subscription, OA = open access.

**Table 3:** Journals with top 100 articles, ranked according to the country of the corresponding author.

Rank	Country of the Corresponding Author	Number of Articles
1	USA	49
2	UK	20
3	Canada	7
4	The Netherlands	4
5	Spain	3
6	Germany	3
7	Sweden	3
8	France	2
9	China	2
9	Finland	2
11	Slovenia	1
12	Switzerland	1
13	Brazil	1
14	India	1
15	Australia	1

**Table 4:** Numbers of articles with top 100 AASs according to neurology subspecialties.

Subspecialty	Number of Articles
Degenerative disorders	32
Neuroscience	23
Stroke	11
Sleep	6
Infection	6
Trauma	5
Headache	4
Demyelinating disorders	3
Seizure	2
Neuromuscular disorders	2
Neuro-oncology	2
Toxicity	1
Pain	1
Movement	1
Genetics	1

**Table 5:** Numbers of articles with top 100 AASs according to subject categories.

Subject Category	Number of Articles
Disease information	29
Lifestyle and disease	27
Science information	19
New or advanced treatments	18
Side effects of treatment	7

## Data Availability

Data cannot be shared because there are embargoes on datasets. Anonymized data will be shared by request from any qualified investigators.

## References

[1] MacRoberts M.H., MacRoberts B.R. (1989). Problems of citation analysis: A critical review. J. Am. Soc. Inf. Sci..

[2] Kim J.E., Kim J.K., Park K.M., Kim Y., Yoon D.Y., Bae J.S. (2016). Top-100 cited articles on Guillain-Barre syndrome: A bibliometric analysis. J. Peripher. Nerv. Syst..

[3] Cline R.J., Haynes K.M. (2001). Consumer health information seeking on the Internet: The state of the art. Health Educ. Res..

[4] Trueger N.S., Thoma B., Hsu C.H., Sullivan D., Peters L., Lin M. (2015). The altmetric score: A new measure for article-level dissemination and impact. Ann. Emerg. Med..

[5] Ruan Q.Z., Chen A.D., Cohen J.B., Singhal D., Lin S.J., Lee B.T. (2018). Alternative metrics of scholarly output: The relationship among altmetric score, Mendeley reader score, citations, and downloads in plastic and reconstructive surgery. Plast. Reconstr. Surg..

[6] Delli K., Livas C., Spijkervet F.K.L., Vissink A. (2017). Measuring the social impact of dental research: An insight into the most influential articles on the Web. Oral Dis..

[7] Peoples B.K., Midway S.R., Sackett D., Lynch A., Cooney P.B. (2016). Twitter predicts citation rates of ecological research. PLoS ONE.

[8] Amath A., Ambacher K., Leddy J.J., Wood T.J., Ramnanan C.J. (2017). Comparing alternative and traditional dissemination metrics in medical education. Med. Educ..

[9] Ortega J.L. (2018). Disciplinary differences of the impact of altmetric. FEMS Microbiol. Lett..

[10] Von Elm E., Altman D.G., Egger M., Pocock S.J., Gøtzsche P.C., Vandenbroucke J.P. (2007). The Strengthening the Reporting of Observational Studies in Epidemiology (STROBE) statement: Guidelines for reporting observational studies. Ann. Intern. Med..

[11] Brigham T.J. (2014). An introduction to altmetrics. Med. Ref. Serv. Q..

[12] Statista, the Statistics Portal. https://www.statista.com/topics/1164/social-networks/.

[13] Williams S.S. (2016). The terrorist inside my husband’s brain. Neurology.

[14] Mez J., Daneshvar D.H., Kiernan P.T., Abdolmohammadi B., Alvarez V.E., Huber B.R., Alosco M.L., Solomon T.M., Nowinski C.J., McHale L. (2017). Clinicopathological evaluation of chronic traumatic encephalopathy in players of American football. JAMA.

[15] Morris M.C., Wang Y., Barnes L.L., Bennett D.A., Dawson-Hughes B., Booth S.L. (2018). Nutrients and bioactives in green leafy vegetables and cognitive decline: Prospective study. Neurology.

[16] Chen H., Kwong J.C., Copes R., Tu K., Villeneuve P.J., van Donkelaar A., Hystad P., Martin R.V., Murray B.J., Jessiman B. (2017). Living near major roads and the incidence of dementia, Parkinson’s disease, and multiple sclerosis: A population-based cohort study. Lancet.

[17] Topiwala A., Allan C.L., Valkanova V., Zsoldos E., Filippini N., Sexton C., Mahmood A., Fooks P., Singh-Manoux A., Mackay C.E. (2017). Moderate alcohol consumption as risk factor for adverse brain outcomes and cognitive decline: Longitudinal cohort study. BMJ.

[18] Richards B.A., Frankland P.W. (2017). The persistence and transience of memory. Neuron.

[19] Siclari F., Baird B., Perogamvros L., Bernardi G., LaRocque J.J., Riedner B., Boly M., Postle B.R., Tononi G. (2017). The neural correlates of dreaming. Nat. Neurosci..

[20] Mlakar J., Korva M., Tul N., Popovic M., Poljsak-Prijatelj M., Mraz J., Kolenc M., Resman Rus K., Vesnaver Vipotnik T., Fabjan Vodusek V. (2016). Zika virus associated with microcephaly. N. Engl. J. Med..

[21] Cao-Lormeau V.M., Blake A., Mons S., Lastere S., Roche C., Vanhomwegen J., Dub T., Baudouin L., Teissier A., Larre P. (2016). Guillain-Barre Syndrome outbreak associated with Zika virus infection in French Polynesia: A case-control study. Lancet.

